# Effects of thrombolysis on outcomes of patients with deep venous thrombosis: An updated meta-analysis

**DOI:** 10.1371/journal.pone.0204594

**Published:** 2018-09-25

**Authors:** Zhenhua Xing, Liang Tang, Zhaowei Zhu, Xinqun Hu

**Affiliations:** Department of Cardiovascular Medicine, The Second Xiangya Hospital, Central South University, Changsha, Hunan, China; Medical University Innsbruck, AUSTRIA

## Abstract

**Background:**

Small randomized controlled studies and meta-analyses have shown that thrombolysis, especially catheter-directed thrombolysis, can reduce the incidence of post-thrombotic syndrome (PTS). However, the recent ATTRACT trial did not demonstrate the same effects. Given this confusing situation, we performed an updated meta-analysis of randomized controlled trials (RCTs) to evaluate the effects of thrombolysis, especially catheter-directed thrombolysis, on the outcomes of deep venous thrombosis (DVT).

**Methods:**

We searched PubMed, Embase, and the Cochrane Library for relevant studies comparing thrombolysis in combination with anticoagulation and with anticoagulation alone. The primary endpoint was PTS during the longest follow-up period. The safety endpoint was the incidence of major bleeding events. We also evaluated the outcomes of catheter-directed thrombolysis as a subgroup analysis.

**Results:**

Six RCTs, including 1418 patients with DVT, were included in our meta-analysis. Thrombolysis in combination with anticoagulation did not reduce PTS (RR: 0.90, [0.80–1.01], *P* = 0.19) and increased major bleeding (RR: 2.07, [1.12–3.81], *P* = 0.02). However, trial sequential analysis (TSA) showed that more patients are needed to support the conclusion that thrombolysis in combination with anticoagulation increased major bleeding. Catheter-directed thrombolysis did not reduce the incidence of PTS (RR: 0.88, [0.68–1.13], *P* = 0.31) and did increase the incidence of major bleeding events (RR: 1.89, [1.00–3.59], *P* = 0.05).

**Conclusion:**

Thrombolysis, including catheter-directed thrombolysis, did not reduce the incidence of PTS and increased the incidence of major bleeding. However, the results were not supported by TSA and sensitivity analysis, so more relevant studies are needed.

## Background

The weighted mean incidence of deep venous thrombosis (DVT) is 5 per 10,000 each year, and this increases significantly with age from about 2–3/10,000 for persons aged 30–49 to 20/10,000 for persons aged 70–79.[1] Approximately half of these patients will develop post-thrombotic syndrome (PTS) despite individualized anticoagulant treatment.[2] About 5–10% of patients with symptomatic DVTs develop severe PTS, which results in limb pain, ulcers, and swelling, and impairs quality of life.[3] Small randomized controlled studies and meta-analyses have shown that thrombolysis, especially catheter-directed thrombolysis, can reduce the incidence of PTS and improve quality of life in patients with DVT.[4–7] However, most trials included in a previous meta-analysis took place before 2000.[7] The bleeding criteria, warfarin treatment, and thrombolytic therapies have changed greatly, which renders the conclusions drawn from that meta-analysis unconvincing. The recent Acute Venous Thrombosis: Thrombus Removal with Adjunctive Catheter-Directed Thrombolysis (ATTRACT) trial demonstrated that the addition of catheter-directed thrombolysis to anticoagulation did not reduce the incidence of PTS and increased major bleeding events. However, because of the low incidence of major bleeding (1%, *P* = 0.049), the trial had limited power to examine whether catheter-directed thrombolysis increased the incidence of major bleeding. Furthermore, most patients developed major bleeding during anticoagulation therapy rather than thrombolysis treatment. Given the contradictory conclusions of previous studies, we performed this updated meta-analysis of randomized controlled trials (RCTs) to evaluate the effects of thrombolysis, especially catheter-directed thrombolysis, on the outcomes of DVT.

## Methods

### Search strategy and selection criteria

This meta-analysis was performed in accordance with the Preferred Reporting Items for System Reviews and Meta-Analyses Statement (PRISMA).[8] We searched PubMed, Embase, and the Cochrane Library for relevant studies published between 1 January 2000 and 10 December 2017, arbitrarily. The keywords used for searching were “iliofemoral/lower extremity,” “thrombosis,” “thromboembolism,” “(deep vein thrombosis) DVT,” “thrombolysis,” and “fibrinolysis.” MeSH, Emtree, and keyword search terms were used in combination. We also used filters to identify RCTs in PubMed and Embase. Results were limited to trials published in English (S1 Table). We manually searched the reference lists of relevant studies and reviews, editorials, and letters to identify further articles. We used Endnote (Thompson ISI ResearchSoft, Philadelphia, PA, US) to manage relevant articles and remove duplicate articles.

### Study criteria, quality assessment, and data extraction

Studies were included if they met the following criteria: (1) the study design was an RCT; (2) all patients were with DVT; (3) patients were randomly assigned to a thrombolysis in combination with anticoagulation group or an anticoagulation-only group; (4) relevant data was retrievable; and (5) the studies were published after 1 January 2000. When relevant data were missing, authors were contacted by e-mail. If that was unsuccessful, references were excluded on account of inaccessibility of data.

The primary endpoint was PTS during the longest follow-up period. The definition of PTS was defined by each study. The safety endpoint was the incidence of major bleeding events. Thrombolysis in Myocardial Infarction (TIMI) major criteria, type 3 or type 5 bleeding according to The Bleeding Academic Research Consortium (BARC), or the Global Utilization of Streptokinase and t-PA for Occluded Coronary Arteries (GUSTO) severe criteria were defined as major bleeding events. We also evaluated the outcomes of catheter-directed thrombolysis as a subgroup analysis. We assessed study quality by evaluating trial procedures for random sequence generation (selection bias), allocation concealment (selection bias), blinding of participants and personnel (performance bias), blinding of outcome assessment (detection bias), and incomplete outcome data (attrition bias). The Cochrane Reviewer’s Handbook 4.2 was used to assess the risk of bias.

Relevant data were collected by 2 independent investigators (L Tang and XF Peng). Disagreements were resolved by discussion between them or a third investigator (XQ Hu). We abstracted the following materials from the selected trials: first author, publication date, study design, characteristics of included participants, total number and events of thrombolysis in combination with anticoagulation group and anticoagulation-only group, thrombolysis and anticoagulation strategies, duration of follow-up, primary study endpoints, and other key outcomes.

### Data analysis

A cumulative relative risk (RR) was calculated by pooling the reported event frequencies from the included RCTs for PTS and major bleeding. Statistical heterogeneity among the trial-specific RRs was checked and quantified by the I^2^ statistic, with I^2^<50% considered low and I^2^>50% high. When low statistical heterogeneity was identified, we preferred a fixed-effect model; otherwise, a random effects model was used. Data analysis was performed on an intention-to-treat basis. We performed sensitivity analysis to assess the contribution of each study to the pooled estimation by excluding one trial at a time and recalculating the pooled RR estimation for the remaining studies. Publication bias was not performed because of the small number of included studies. All analysis was performed using Review Manager Software (Review Manager (RevMan) [Computer program]. Version 5.3. Copenhagen: the Nordic Cochrane Centre, the Cochrane Collaboration, 2014).

### Trial sequential analysis (TSA)

Cumulative meta-analyses are prone to produce type I and type II errors because of repeated testing of significance as trial data accumulates. Statistically significant small trials are often overlooked when contradictory results from adequately powered and bias-protected trials emerge.[9,10] TSA is similar to interim analyses in a single trial, where monitoring boundaries are used to determine whether a trial can be terminated early when a *P* value is sufficiently small to show the anticipated effect. Analysis was performed using Trial Sequential Analysis Viewer (0.9.5.9 Beta) anticipating a 25% relative risk reduction for efficacy outcome, α = 5% and 1−β = 80%, and estimating the required diversity-adjusted information size. This methodology is described in detail elsewhere.[11,12]

## Outcomes

### Search results and bias assessment

**Fig 1:**
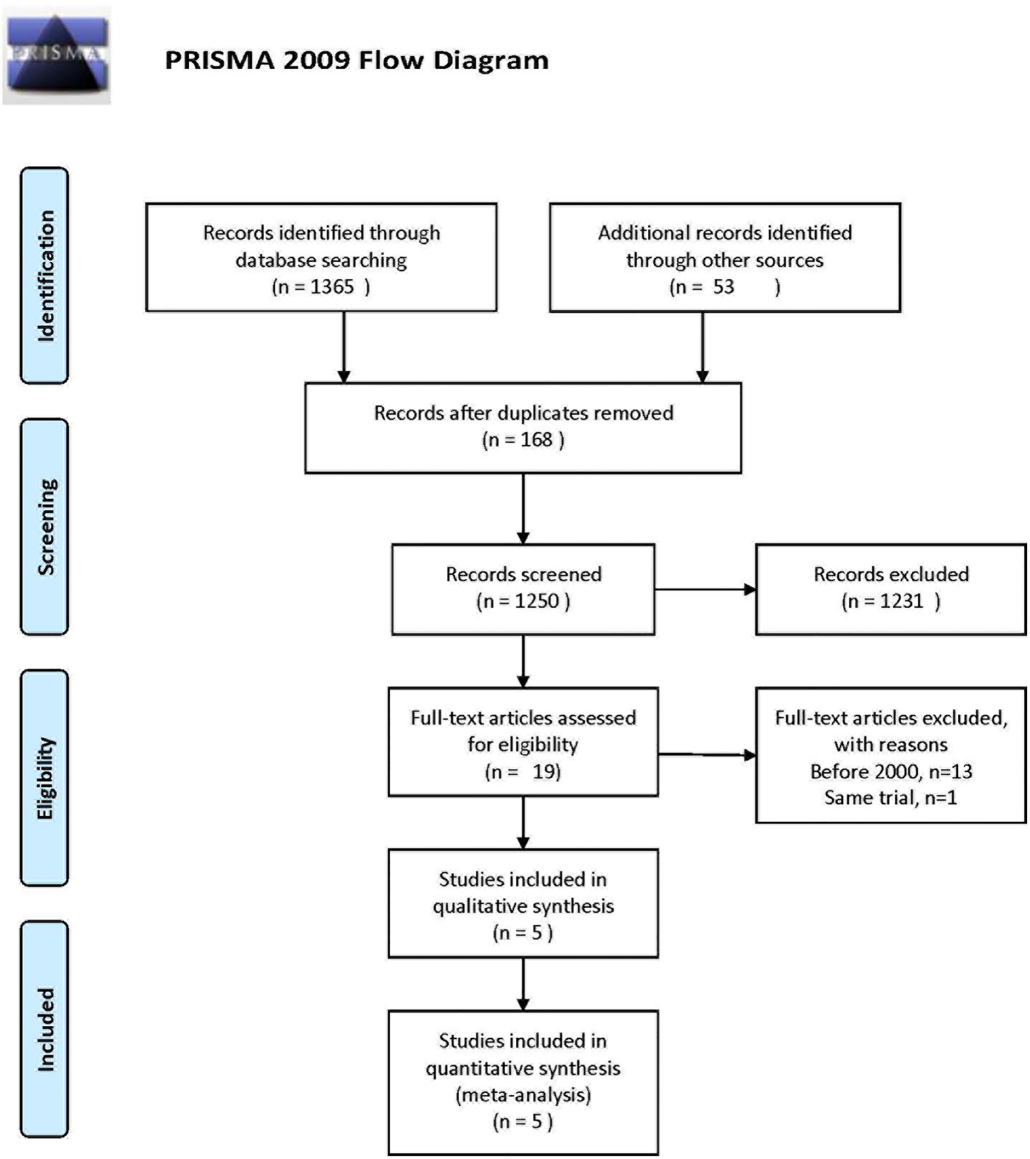
Flow diagram of literature searched for review.

As depicted in Fig 1, our combined search strategies identified 1418 potential relevant studies. After more detailed evaluation, we identified 19 RCTs. We excluded 13 RCTs published before January 1, 2000, none of which indicated the incidence of PTS and all of which had different criteria for bleeding events and warfarin treatment adjustment compared with current criteria. Finally, 6 RCTs, including 1365 patients with DVT, were included in our meta-analysis.[5,6,13–15] The characteristics of these included RCTs are shown in Table 1. Among them, four studies were multicenter studies (Schweizer 2000, Enden 2009, Enden 2012, Vedantham 2017). Clinical heterogeneity was mostly attributable to different times of symptom onset, clinical characteristics of included patients, different strategies of thrombolysis, and duration of follow-up. Four RCTs evaluated the effectiveness of catheter-directed thrombolysis. Among them, three studies even performed angioplasty or stent implantation. One RCT evaluated locoregional or systemic thrombolysis in combination with anticoagulation compared with anticoagulation alone. We only extracted the data of locoregional thrombolysis for our meta-analysis because of the higher rate of bleeding in the systemic thrombolysis group. Thrombolytic drugs including streptokinase, alteplase, urokinase, and recombinant tissue plasminogen activator varied greatly (rt-PA) (Table 2). Most studies chose warfarin as the optimal anticoagulant, except for Vedantham 2017, which chose rivaroxaban. Compression stockings were used in 3 studies (Enden 2012, Schweizer 2000, Vedantham 2017). Follow-up time varied from 6 months to 24 months. We used the Cochrane Reviewer’s Handbook 4.2 to assess risk of bias (S1 Fig). No high-risk studies existed. Three of them had a low risk of bias.

**Table 1:** Detailed characteristics of included studies.

Study	Study design	Country	Age	Male (%)	Onset of symptoms	Follow-up
**Schweizer 2000**	Multicenter	Germany	40	43	5.6 d	12 m
**Elsharawy 2002**	Single-center	Egypt	47	69	4.5 d	6 m
**Enden 2009**	Multicenter	Norway	52	32	6.4 d	6 m
**Enden 2012**	Multicenter	Norway	52	37	6.6 d	24 m
**Ugurlu 2002**	Single-center	Turkey	48	62	5 d	-
**Vedantham 2017**	Multicenter	America	52.5	61.5	-	24 m

**Table 2:** Thrombolysis and anticoagulation strategies.

Study	Interventions	
	Thrombolysis+anticoagulation	Anticoagulation
Elsharawy 2002	Thrombolysis with catheter using streptokinase, (pulse spray 1000, 000 U/h), then 100, 000 U/h until complete lysis; warfarin.	Warfarin
Enden 2009	Alteplase 0.01 mg/kg/h, with a maximal dose of 20 mg per 24 h and maximal duration of 96 h. LMWH given twice daily was initiated 1 h after removal of catheters.	Warfarin (INR2-3)
Enden 2012	Before catheter-based thrombolysis, LMWH for 5 d, catheter-based thrombolysis with alteplase and unfractionated heparin (IV), with or without angioplasty or stents; then warfarin (INR2-3); compression treatment.	LMWH and warfarin 5 d; then warfarin alone (INR 2–3); compression treatment
Schweizer 2000	Locoregional tissue plasminogen activator (20 mg/d) or urokinase (100,000 U/d) or systemic streptokinase (3,000,000 U/d) or urokinase (5,000,000 U/d); warfarin (2–3) + compression	Warfarin (2–3) + compression
Ugurlu 2002	Streptokinase 250,000 in 30 min, then 100,000 U/h until 1,500,000 U; then heparin (IV); warfarin (INR) 2 d later	Heparin (5000 bolus+ 1–1500 U/h); warfarin (INR) 2d later
Vedantham 2017	Catheter-based intrathrombus delivery of recombinant tissue plasminogen activator (rt-PA) and thrombus aspiration ormaceration, with or without stenting; rivaroxaban + compression.	Rivaroxaban + compression

## Quantitative data synthesis

### PTS

**Fig 2:**
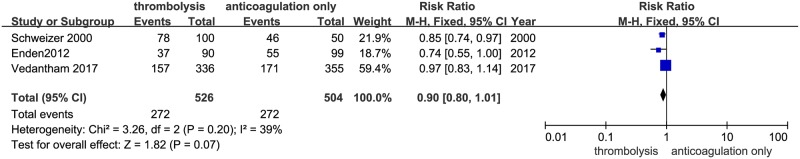
Thrombolysis + anticoagulation group vs. anticoagulation-only group on the outcomes of PTS.

Our analysis showed that thrombolysis in combination with anticoagulation in patients with DVT was not associated with a significant reduction in PTS in the fixed model (272 of 526 [52%] in the thrombolysis+anticoagulation group vs. 244 of 504 [54.0%] in the anticoagulation-only group, RR: 0.90, [0.80–1.01], *P* = 0.19, I^2^ = 39%, Fig 2). Sensitivity analysis was performed by excluding one trail at a time and recalculating the pooled RR for the remaining trials, which found that the most recent study (Vedantham 2017) affected the result. When we excluded this study, the results became positive (RR: 0.80, [0.68–0.93]). Sensitivity analysis showed that this result is not fairly reliable.

### Major bleeding

**Fig 3:**
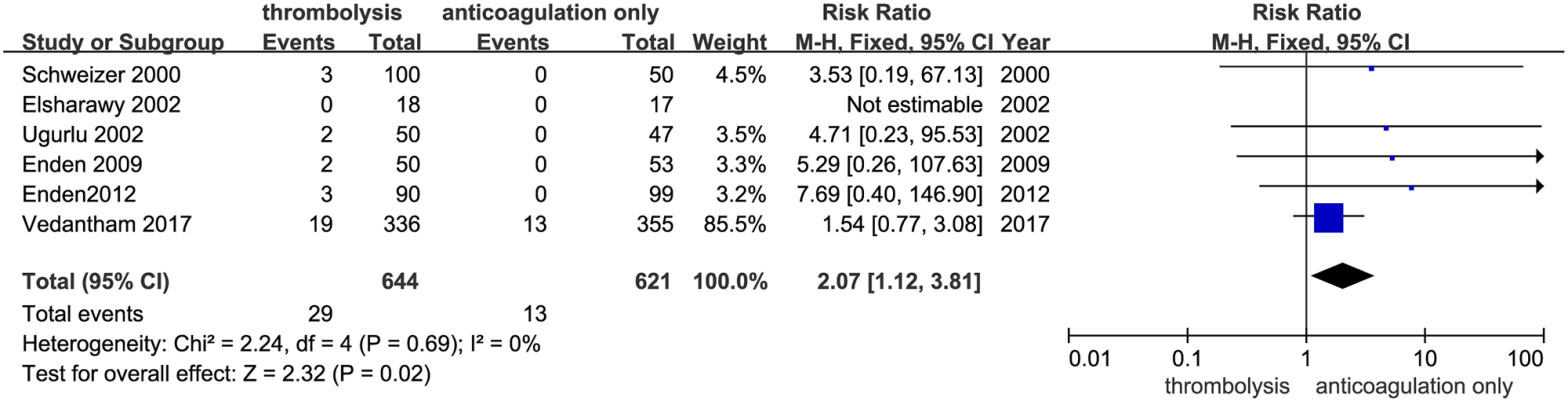
Thrombolysis + anticoagulation group vs. anticoagulation-only group on the outcomes of major bleeding.

**Fig 4:**
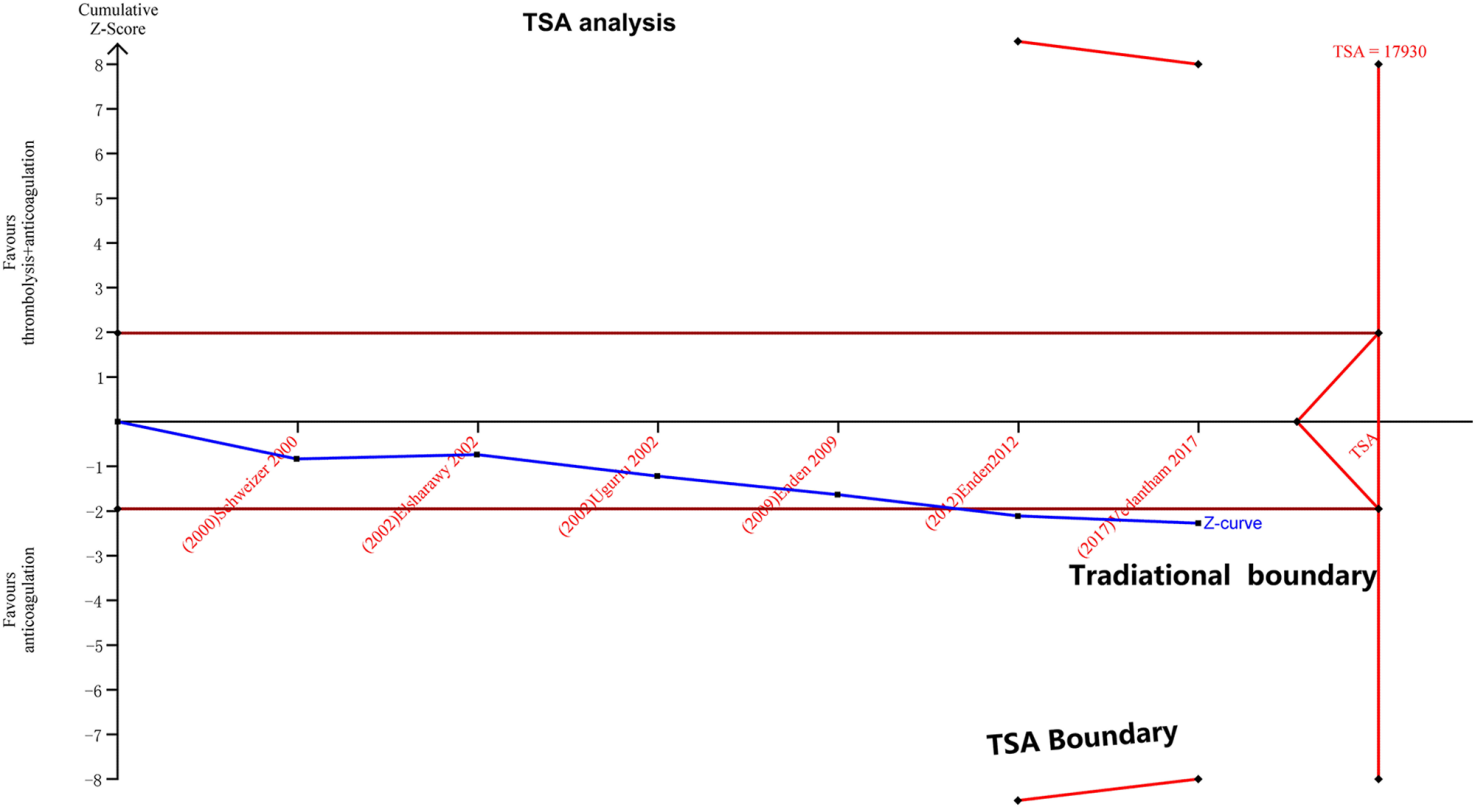
Trial sequential analysis (TSA) for the outcome of major bleeding. The cumulative Z-curve crossed the traditional boundary (*P* = 0.05) but not the TSA boundary, indicating a lack of firm evidence for a 25% reduction in major bleeding with anticoagulation only compared with thrombolysis in combination with anticoagulation. The required sample size is based on an anticipated intervention effect of a 25% relative risk reduction, a control event proportion estimated from the cumulative traditional event proportion, and a diversity of 25%, α = 0.05, and β = 0.20.

Thrombolysis in combination with anticoagulation increased the incidence of major bleeding in the fixed effect model (29 of 644 [4.5%] in the thrombolysis in combination with anticoagulation group vs. 13 of 621 [2.1%] in the anticoagulation-only group, RR: 2.07, [1.12–3.81], *P* = 0.02, I^2^ = 0%, Fig 3). Sensitivity analysis showed that no studies affected the overall effect. However, in the TSA, the cumulative Z-curve crossed the traditional boundary (*P* = 0.05) but not the TSA boundary, indicating a lack of a firm evidence (α = 5%, 1-β = 80%) in increasing major bleeding with thrombolysis in combination with anticoagulation compared with anticoagulation only (Fig 4). Because of the low incidence of major bleeding, more clinical trials are needed to verify this result.

### Catheter-directed thrombolysis

**Fig 5:**
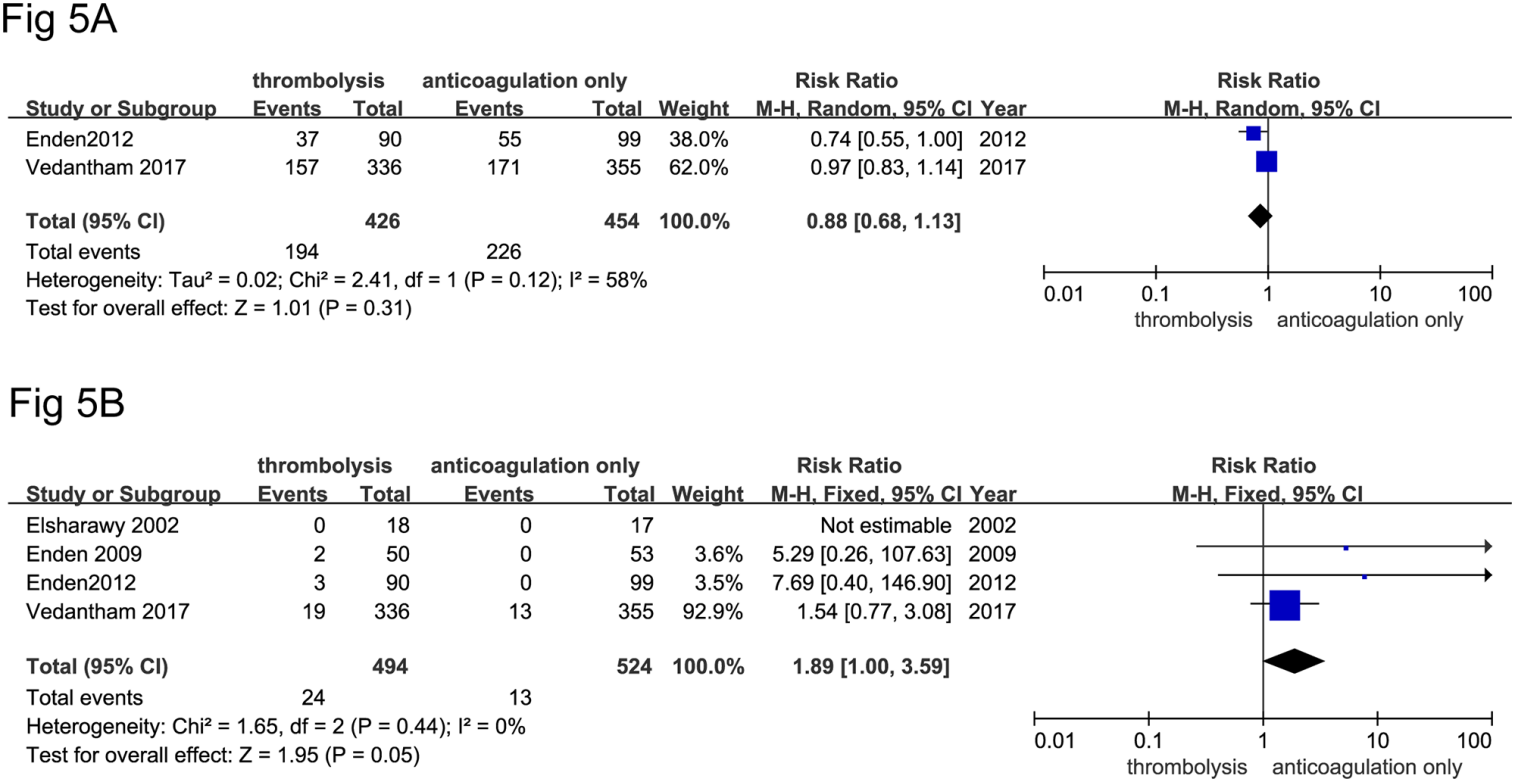
A) catheter-directed thrombolysis + anticoagulation group vs. anticoagulation-only group on the outcomes of PTS; B) catheter-directed thrombolysis + anticoagulation group vs. anticoagulation-only group on the outcomes of major bleeding.

Catheter directed-thrombolysis did not reduce the incidence of PTS in the random effects model (194 of 426 [45.5%] in the thrombolysis in combination with anticoagulation group vs. 226 of 454 [49.7%] in the anticoagulation-only group, RR: 0.88, [0.68–1.13], *P* = 0.31, I^2^ = 58%, Fig 5A). At the same time, catheter directed-thrombolysis did increase the incidence of major bleeding events in the fixed model (24 of 494 [4.85%] in the thrombolysis in combination with anticoagulation group vs. 13 of 524 [2.5%] in the anticoagulation-only group, RR: 1.89, [1.00–3.59], *P* = 0.05, I^2^ = 0%, Fig 5B).

## Discussion

Our meta-analysis and TSA demonstrated that thrombolysis in combination with anticoagulation therapy did not reduce the incidence of PTS compared with anticoagulation alone. Thrombolysis in combination with anticoagulation might increase the incidence of major bleeding. However, because of the low incidence of major bleeding, more RCTs are needed.

Although anticoagulation therapy with warfarin or novel oral anticoagulants (NOACs) and compression stocking are the main treatments for DVT, approximately half of patients with DVT will develop PTS.[5] However, a meta-analysis with 1462 patients did not demonstrate that patients with DVT could benefit from compression stockings.[16] More effective treatments are needed to deal with DVT. As a result, thrombolysis, especially catheter-directed thrombolysis, is regarded as a last resort. Although many studies have demonstrated that thrombolysis can effectively dissolve the thrombus and improve venous patency, most of them were performed before 2000.[17] PTS was often overlooked in earlier studies. These single-center studies with limited patients often showed far better outcomes by systemic thrombolysis. Selection bias, performance bias, detection bias, and reporting bias are usually inevitable. There is no doubt that systemic thrombolysis increases the incidence of major bleeding. Bleeding criteria and anticoagulant strategies with warfarin have developed considerably since these earlier studies were performed. All of this renders these studies inadequate to direct current clinical practice. Our meta-analysis, which summarized recent relevant studies, does not support routine thrombolysis or even catheter-directed thrombolysis. Our conclusion differs from that of previous clinical trials and meta-analyses because of the inclusion of the recent ATTRACT trial.[5,7]

Thrombolysis is an effective means of restoring the patency of deep veins and avoiding recurrence. The recognized risk factors for DVT include age, male gender, cancer, surgery, and similar factors [18], most of which cannot be resolved by thrombolysis. Nevertheless, surgical patients with DVT are at a lower risk of recurrence than patients with other unchangeable risk factors.[19] Patients with transient risk factors may benefit from thrombolysis, especially catheter-directed thrombolysis. However, patients with unchangeable risk factors are at higher risk of recurrence. Unfortunately, none of these RCTs distinguished these patients with transient risk factors from patients with unchanged risk factors. This may be why thrombolysis failed to reduce the incidence of PTS.

Thrombolytic therapy increased the incidence of major bleeding, which was not supported by TSA analysis. Because of the low incidence of major bleeding, RCTs with larger sample sizes are needed. The trend in which thrombolysis increases the incidence of major bleeding is in accordance with previous RCTs and meta-analyses.[5,7] Our meta-analysis included the Schweizer 2000 and Ugurlu 2002 trials, in which systemic thrombolysis increased major bleeding considerably.[20] However, catheter-directed thrombolysis had a lower incidence of major bleeding than systemic thrombolysis.[7] Most bleeding complications of catheter-directed thrombolysis took place at the puncture site, and cases of major bleeding (e.g., intracranial hemorrhage) were a small minority.[21] As a result, proper treatment of the puncture site is extremely important.

## Conclusion

Thrombolysis, including catheter-directed thrombolysis, did not reduce the incidence of PTS and increased the incidence of major bleeding. More relevant studies are needed.

## Supporting information

S1 Table Full electronic search strategy for PubMed. (DOCX)

S1 Fig Bias assessment using Cochrane Reviewer’s Handbook 4.2. (TIF)

S1 Checklist PRISMA checklist. (DOC)
