# Supplementary material for: Effects of thrombolysis on outcomes of patients with deep venous thrombosis: An updated meta-analysis
Source: PLoS One. 2018 Sep 25;13(9):e0204594. doi: 10.1371/journal.pone.0204594 (PMC6155544; doi:10.1371/journal.pone.0204594)
Supplement: S1 Table — (DOCX) [file pone.0204594.s001.docx]

**S1 Table: Full electronic search strategy for PubMed**

| Search | Search Query | Items found |
| --- | --- | --- |
| #1 | Deep Vein Thrombosis[MeSH Terms] | 51369 |
| #2 | DVT[Title/Abstract | 9037 |
| #3 | thromboembolism[MeSH Terms] | 51389 |
| #4 | thrombosis[MeSH Terms] | 121043 |
| #5 | lower extremity[MeSH Terms] | 154331 |
| #6 | iliofemoral[Title/Abstract] | 1794 |
| #7 | thrombolysis[Title/Abstract] | 22751 |
| #8 | fibrinolysis[MeSH Terms] | 20648 |
| #9 | ("2000"[Date - Publication] : "2017"[Date - Publication]) | 14568788 |
| #10 | #3 OR #4 | 163934 |
| #11 | #5 OR #6 | 155910 |
| #12 | #7 OR #8 | 42681 |
| #13 | #11 AND #12 | 7837 |
| #14 | #1 OR #2 OR #13 | 57264 |
| #15 | #12 AND #14 | 2346 |
| #16 | #9 AND 15 | 1305 |
| #17 | (clinical[tiab] AND trial[tiab]) OR "clinical trials as topic"[mesh] OR "clinical trial"[pt] OR random*[tiab] OR "random allocation"[mesh] OR "therapeutic use"[sh] | 5006655 |
| #18 | #16 AND #17 | 836 |
