# Supplementary figures and images for: Effects of thrombolysis on outcomes of patients with deep venous thrombosis: An updated meta-analysis
Source: PLoS One. 2018 Sep 25;13(9):e0204594. doi: 10.1371/journal.pone.0204594 (PMC6155544; doi:10.1371/journal.pone.0204594)

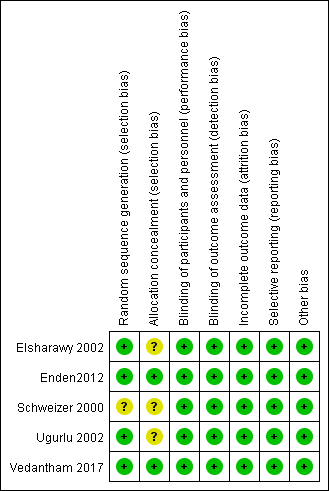

Supplement: S1 Fig — (TIF) [file pone.0204594.s002.tif]
